# A mixture of amino acids and other small molecules present in the serum suppresses the growth of murine and human tumors *in vivo*

**DOI:** 10.1002/ijc.27756

**Published:** 2012-08-01

**Authors:** Gyula Kulcsár, Dezső Gaál, Péter I Kulcsár, Ákos Schulcz, Tamás Czömpöly

**Affiliations:** 1 Immunal Ltd., Cancer Research and Product Development LaboratoryH-7630 Pécs, Finn u. 1/1., Hungary; 2 Department of Experimental Pharmacology, National Institute of OncologyH-1122 Budapest, Ráth György u. 7-9., Hungary

**Keywords:** antitumor effect, amino acids, vitamins, apoptosis, chemotherapy

## Abstract

Previously we have hypothesized that the small molecules which are selectively accumulated in cancer cells might participate in a non-immunological antitumor surveillance mechanism. We demonstrated earlier that a mixture of experimentally selected substances (“active mixture”, AM: l-arginine, l-histidine, l-methionine, l-phenylalanine, l-tyrosine, l-tryptophan, l-ascorbate, d-biotin, pyridoxine, riboflavin, adenine, l(-)malate) possesses a selective toxic effect *in vitro* on a variety of tumor cell lines, and we have shown that the AM selectively induces apoptosis of cancer cells *in vitro*. To explore the *in vivo* significance of our earlier findings we examined the antitumor effect of AM in Colon 26 murine colorectal adenocarcinoma, B16 murine melanoma, MXT murine mammary carcinoma, S180 murine sarcoma, P388 murine lymphoid leukemia, HL-60 human promyeloid leukemia, PC-3 human prostate carcinoma, and HT-29 human colon carcinoma tumor models. Treatment of tumor bearing mice with AM inhibited the growth of the tumors investigated, with an inhibitory effect ranging from 40 to 69%. The AM had a comparable antitumor effect with 5-fluorouracil and cisplatin in the Colon-26 tumor model, and combined treatment with AM and 5-fluorouracil or cisplatin resulted in an enhanced tumor growth inhibitory effect. The AM induced apoptosis through the mitochondrial pathway and induced G1 arrest in PC-3 cells and increased the number of apoptotic cells in PC-3 xenografts. These findings suggest that the AM might offer an interesting perspective in the treatment of cancer and in combination with other treatments may offer hope for a more effective cancer therapy.

## Introduction

All multicellular living organisms have different mechanisms that reduce the otherwise high probability of cancer development. In higher vertebrates at least three types of nonimmune surveillance take part in the first line defense against tumors. In addition to the two major forms of surveillance: the genetic (DNA repair, checkpoint control) and the intracellular (largely apoptosis related) surveillance, recent findings suggest the existence of the epigenetic surveillance (stringency of chromatin imprinting).^1^, ^2^ As a result of their mechanisms the role of these first line defense systems is to prevent cancer cell formation.

Cancer cells formed despite the nonimmune safeguards should be eliminated by the next stage of host protection. Although, there are indications for the existence of an intercellular surveillance (microenvironmental control of tumor),^1^, ^2^ the main role in the second line of defense is ascribed to the immune system, the only defense mechanism which has components in the circulatory system. However, accumulating evidence indicates that tumors use multiple mechanisms to evade the effect of the immune surveillance. In addition immunoediting can lead from immune surveillance to tumor escape. Consequently, the immune system frequently fails to eliminate cancer cells, and its action is mainly restricted to the virus induced tumors.^1^–^7^

Considering the above described limitations in the efficiency of immune surveillance in tumor control and the multiple mechanisms used by tumors to evade the effect of the immune system, together with the fact that despite these limitations tumors do not develop in the majority of people, motivated us to examine the possibility that besides the well established immunological and nonimmunological surveillance additional defense mechanism(s) might operate to prevent the development of tumors.

We hypothesized that the components of this additional defense mechanism (a “surveillance”) might be in the circulatory system and turned our attention to those small substances (amino acids, monosacharides, nucleobases, *etc*.) present in the serum which are differentially taken up by tumor and normal cells.^8^ Since the elevated uptake of glucose and increased glycolitic activity of cancer cells has been first reported by Otto Warburg,^9^ it has been shown that in addition to glucose many molecules (amino acids, vitamins) are accumulated in cancer cells.^10^–^12^ The accumulation of these substances by cancer cells is utilized in positron emission tomography,^13^ and targeting strategies has been started to emerge on the basis of amino acid and vitamin accumulation.^14^, ^15^ In recent years it became increasingly clear that there are a significant number of common signaling pathways regulating both cellular metabolism and cell proliferation.^16^ Taking into account that many molecules in the living system have more than one fundamentally different role we assumed that some of the accumulated substances besides their usual role in metabolism might participate in a defense system capable of killing emerging cancer cells.

**What's new?**

Tumor cells have increased glucose uptake and in addition accumulate other molecules such as amino acids and vitamins to higher levels than non-tumor cells. The authors hypothesized that this accumulation represents a tumor defense mechanism and tested the effect of a mixture of these substances called Active Mixture in murine and human xenograft tumor models. Active Mixture induced apoptosis of tumor cells both *in vitro* and *in vivo* and effectively diminished tumor growth in a variety of tumor models offering an interesting new perspective on tumor therapy.

Previously we have substantiated our hypothesis by experimentally selecting substances present in the serum whose mixture (“active mixture,” AM) showed a selective toxic effect *in vitro* on a variety of tumor cell lines.^17^, ^18^ We have also demonstrated by several methods that the AM selectively induce apoptosis of cancer cells *in vitro.*^19^, ^20^ Recently we have shown that combination of the AM with various cytostatic agents or irradiation results in an increased cytotoxic effect *in vitro.*^21^

In this study we investigated the *in vivo* antitumor effect of the AM alone or in combination with cytostatic agents. In this article we provide evidence that the AM has a significant tumor inhibitory effect *in vivo*, treatment with AM increases the antitumor activity of cytostatic agents, and induces apoptosis both *in vitro* and *in vivo*. In addition we demonstrate that the AM induces apoptosis *via* the mitochondrial pathway, and influences the proliferation of cancer cell by inducing G1 arrest.

## Material and Methods

### Materials

The selection of the components of the “active mixture” (AM) and “control mixture” (CM) has been described previously,^17^, ^18^ a brief description is provided in Supporting Information Materials and Methods. The AM used for the *in vivo* experiments was formulated on the basis of the above mentioned results^17^, ^18^ with consideration of unavoidable practical aspects (the rate of excretion, the solubility, the stability, the pharmaceutical grade and the price, *etc*. of the components). This “practical” AM has the following composition: 32.07 mM l(-)-malic acid, 72.64 mM l-phenylalanine, 51.66 mM l-arginine, 73.47 mM l-histidine, 1.38 mM l-tyrosine, 20.11 mM l-methionine, 14.69 mM l-tryptophan, 0.06 mM d-biotin, 1.02 mM pyridoxine hydrochloride, 2.49 mM adenine hydrochloride, 0.41 mM riboflavin-5′-phosphate, and 23.39 mM l-ascorbic acid. The solution was prepared by reconstituting of Culevit powder for solution (manufactured by Human Serum and Pharmaceutical Manufacturing Company, Gödöllő, Hungary for Immunal Ltd., Budapest, Hungary). The CM used for the *in vivo* experiment has the following composition: 32.07 mM succinic acid disodium salt, 72,64 mM l-valine, 51.66 mM l-asparagine, 73.47 mM L-serine, 1.38 mM L-alanine, 20.11 mM glycine, 14.69 mM L-proline, 0.06 mM thiamin hydrochloride, 1.02 mM folic acid sodium salt, 2.49 mM hypoxanthine, 0.41 mM d-pantothenic acid hemicalcium salt, 23.39 mM niacin.

On the basis of a 25-fold *in vivo* dilution factor (200 μl injected mixture/5 ml of extracellular fluid volume^22^) the concentrations of the components of the *in vitro* used AM and CM were calculated with division of the *in vivo* used concentrations by 25.

All chemicals, media, and materials used in this study were purchased from Sigma (Budapest, Hungary) except otherwise indicated.

### Cell lines, tumors and animals

The description of cell lines, tumors and animals is provided in Supporting Information Materials and Methods.

### Evaluation of antitumor activity of the active mixture in syngeneic mouse tumor models

P388 lymphoid leukemia (1 x 10^7^ cells/mouse) were injected subcutaneously (s.c.) into the flank of BD2F1 mice. Tissue fragments (3–4 mm, app. 25 mg in weight) of Colon 26 adenocarcinoma and S180 sarcoma were transplanted s.c. into the flank of BALB/c mice. Tissue fragments of MXT hormone sensitive mammary carcinoma and B16 melanoma were transplanted s.c. into the flank of BD2F1 mice. The treatments were started on the first day after tumor inoculation. The AM was given i.p. daily in a volume of 0.2 ml (in the case of dose dependence experiment 0.2, 0.1 or 0.05 ml) at 1-hr intervals eight times a day for 10 consecutive days (or for 17 days as indicated). Cisplatin was injected i.p. once a day on Days 1, 5 and 9 at a dose of 2.5 mg/kg. 5-FU was administered i.p. once a day for 5 days after tumor inoculation at a dose of 25 mg/kg. Control mice were injected with saline. The tumor growth inhibition (TGI) was monitored by measuring the tumor volume with a digital caliper. Tumor volume (*V*) was calculated by the formula of *V* = *a*^2^ x *b* x π/6 where ”*a*” and ”*b*” stand for the shortest and the longest diameter of the tumor, respectively.^23^ All animal procedures were performed in accordance with published guidelines on the welfare of animals in experimental neoplasia,^24^ therefore the animals were decapitated when the volume of the tumors reached or exceeded ∼2,000 mm^3^. The protocols were approved by the Ethical Committee of Animal Experiments of the National Institute of Oncology, Budapest, Hungary.

### Evaluation of antitumor activity of the active mixture in human xenograft tumor models

Tissue fragments (3–4 mm, app. 25 mg in weight) of HL-60 promyeloid leukemia, PC-3 human prostate carcinoma and HT-29 human colon carcinoma were transplanted s.c. into the intrascapular region of CB17/ICR-*Prkdc*^*scid*^ mice. For the long-term experiments 5x10^5^ PC-3 cells were injected s.c. into the left flank. To spare animals, in the experiments with CM five mice per group (bearing 2 tumors/animal) were treated. The AM and CM was given i.p. daily in a volume of 0.2 ml at 1-hr intervals eight times a day for 10 consecutive days (or for 16 days as indicated). To reduce the potential distress on the animals the dosage regimen was changed in the long-term experiments: the AM was given i.p. daily in a volume of 0.2 ml at 2-hr intervals four times a day for 30 consecutive days. Control mice were injected with saline except for one experiment in which CM was also used. The evaluation of TGI was performed as described for the syngeneic mouse tumor models.

The description of the cell growth assay, annexin V staining, TUNEL assay, measurement of mitochondrial membrane potential and mitochondrial mass, western-blot, cell division tracking, cell cycle analysis and quantitative RT-PCR (QPCR) is provided in Supporting Information Materials and Methods.

### Statistical analysis

Statistical analysis was performed by either unpaired Student's *t*-test or ANOVA followed by Bonferroni test, as indicated. *p* values less than 0.05 were considered statistically significant. Statistical analyses were performed with OriginPro7 software.

## Results

### Antitumor activity of the AM in various mouse syngeneic and human xenograft tumor models

**Figure 1:**
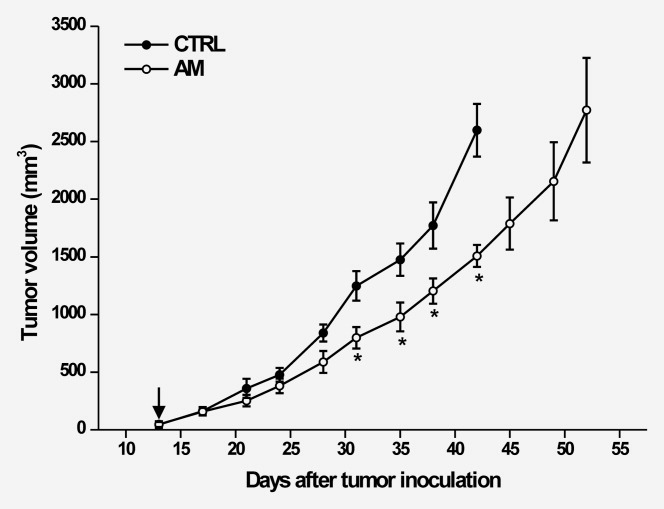
The tumor growth inhibitory effect of the AM is sustainable during the long term treatment of PC-3 xenografts. Mice were treated with AM started from the 13^th^ day after tumor inoculation (arrow). Error bars represent SEM. *p < 0.05 (Student's *t*-test).

We studied the antitumor activity of the AM on various syngeneic mouse tumor models (Colon 26 adenocarcinoma, B16 melanoma, MXT breast carcinoma, S180 sarcoma, P388 lymphoid leukemia) and on human xenograft tumor models (HL-60 human promyeloid leukemia, the PC-3 human prostate carcinoma and the HT-29 human colon carcinoma). In case of the syngeneic mouse models treatment with AM for 10 days produced a significant growth inhibitory effect ranging from 55 to 69%. The growth of the human xenograft tumors was also significantly inhibited (TGI: 40%) (Table 1). On the basis of body weight measurements performed on every second day we observed no toxic effect (data not shown). Using the P388 lymphoid leukemia model the antitumor activity of the AM was found to be dose dependent and could be sustained during an extended treatment period (Supporting Information Fig. S1). We also examined the effect of chronic AM exposure using the PC-3 tumor model. In these experiments we used cells instead of tissue fragments for tumor inoculation and we changed the dosage regimen (0.2 ml of the AM was given i.p. daily at 2-hr intervals four times a day). According to our results the TGI was lower at the early phase of the long-term experiments than in our previous short term experiments; however, the TGI measured at the end of the experiment reached the same level as in the short term experiments (TGI at day 42: 41%, Fig. 1). To exclude any potential nonspecific effect we performed an experiment with a control mixture (CM) which had the same osmolarity as the AM and contained similar but ineffective small molecules as it is described in the “Supporting Information Materials” section. We have found that the CM had no effect on the growth of PC-3 xenografts (Supporting Information Fig. S2).

**Table 1:** Antitumor activity of the AM in mouse syngeneic and human xenograft tumor models

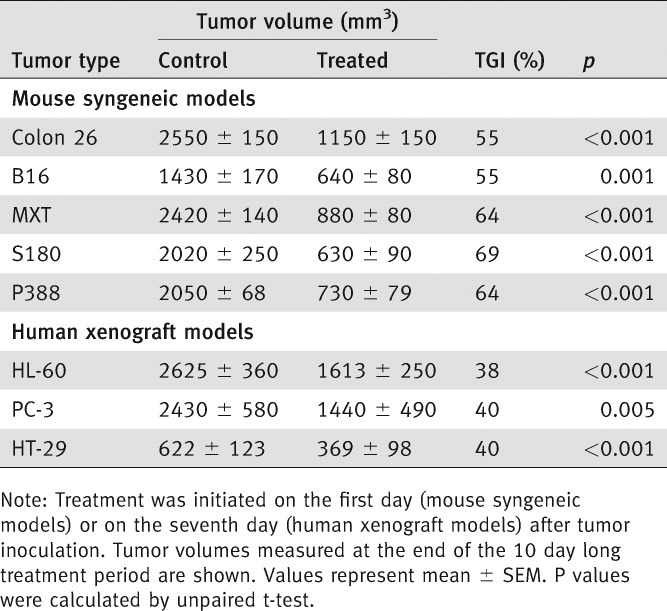

### The antitumor effect of the AM is comparable with 5-FU in the Colon 26 colon carcinoma model

**Figure 2:**
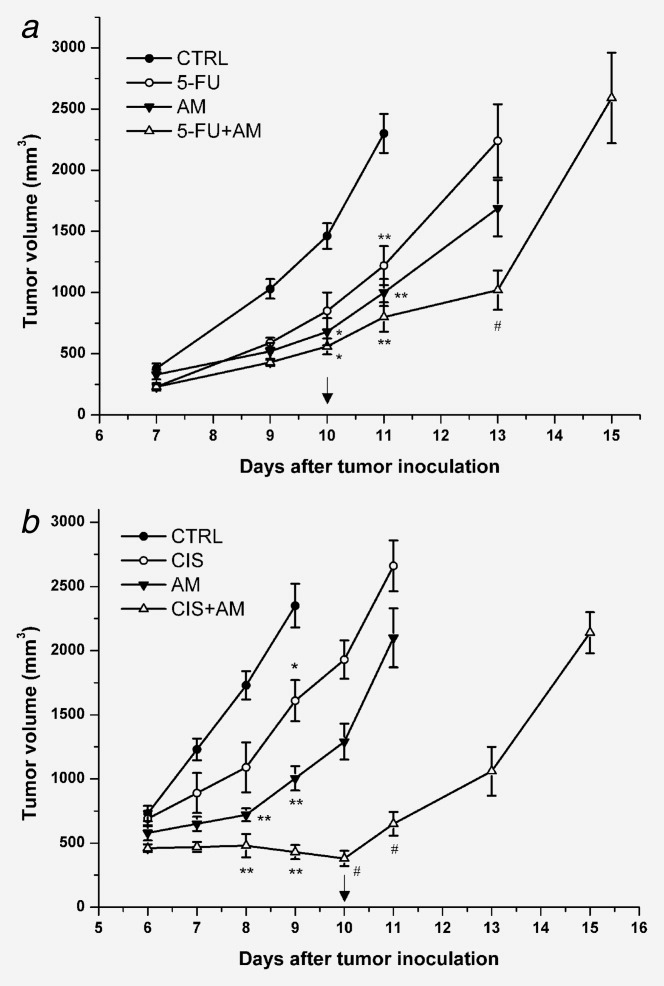
Combination of the AM with 5-FU or cisplatin increases the TGI in the Colon 26 colon carcinoma model. (*a*) Mice were treated with 5-FU, with AM, or with 5-FU and AM. *p < 0.05, **p < 0.001 *vs.* control; #*p* < 0.001 *vs*. 5-FU (ANOVA). *B*, Mice were treated with cisplatin, with AM, or with cisplatin and AM. *p < 0.05, **p < 0.001 *vs*. control; #*p* < 0.001 *vs.* cisplatin and *vs.* AM (ANOVA). Arrows indicate the last days of treatment. Error bars represent SEM.

We have found that treatment of the Colon 26 adenocarcinoma with the AM or 5-FU inhibited the tumor growth with comparable efficacy. The TGI at the end of the 10 day long treatment period was 57% and 47% (*p* < 0.001 for both) for the AM and 5-FU, respectively. Concomitant administration of the AM and 5-FU produced a 65% TGI (*p* < 0.001 *vs*. control, Fig. 2*a*). At the end of the treatment period tumor volumes of the group which received combined treatment showed no statistically significant difference when compared with groups treated with 5-FU alone or the AM alone. However, a sustained TGI was observed in the combined treatment group after the termination of treatment, and the difference between the tumor volumes of the group treated with 5-FU only and the group which received the combined treatment became significant.

### Combination of the AM and cisplatin increases the TGI in the Colon 26 colon carcinoma model

Next we assessed the antitumor effect of the AM in comparison with cisplatin. Treatment of Colon 26 adenocarcinoma with AM seemed to produce a higher TGI than cisplatin (AM: 57%, *p* < 0.001 *vs.* control, cisplatin: 31%, *p* < 0.001 *vs.* control), however the difference between these treatment groups was not significant. Combination of the AM with cisplatin resulted in a 73% (*p* < 0.001) TGI (Fig. 2*b*). It is important to note that significant difference was observed between the antitumor effect of the single and combined treatments. Moreover, in case of the combined treatment the tumor volume decreased during the treatment period, and a statistically significant TGI was maintained even after treatment termination.

### The AM induces apoptosis of PC-3 cells both *in vitro* and *in vivo*

We sought to investigate the mechanism underlying the *in vivo* antitumor activity of the AM. We have selected the PC-3 human androgen independent prostate carcinoma model for further *in vitro* and *in vivo* investigations. To rule out any potential nonspecific effect we applied a CM corresponding to the composition of the *in vitro* used AM in all *in vitro* experiments.

In a series of *in vitro* experiments we have found that treatment with AM, but not with CM inhibits the growth of PC-3 cells and induces apoptosis as evidenced by phosphatidylserine externalization (Supporting Information Fig. S3).

**Figure 3:**
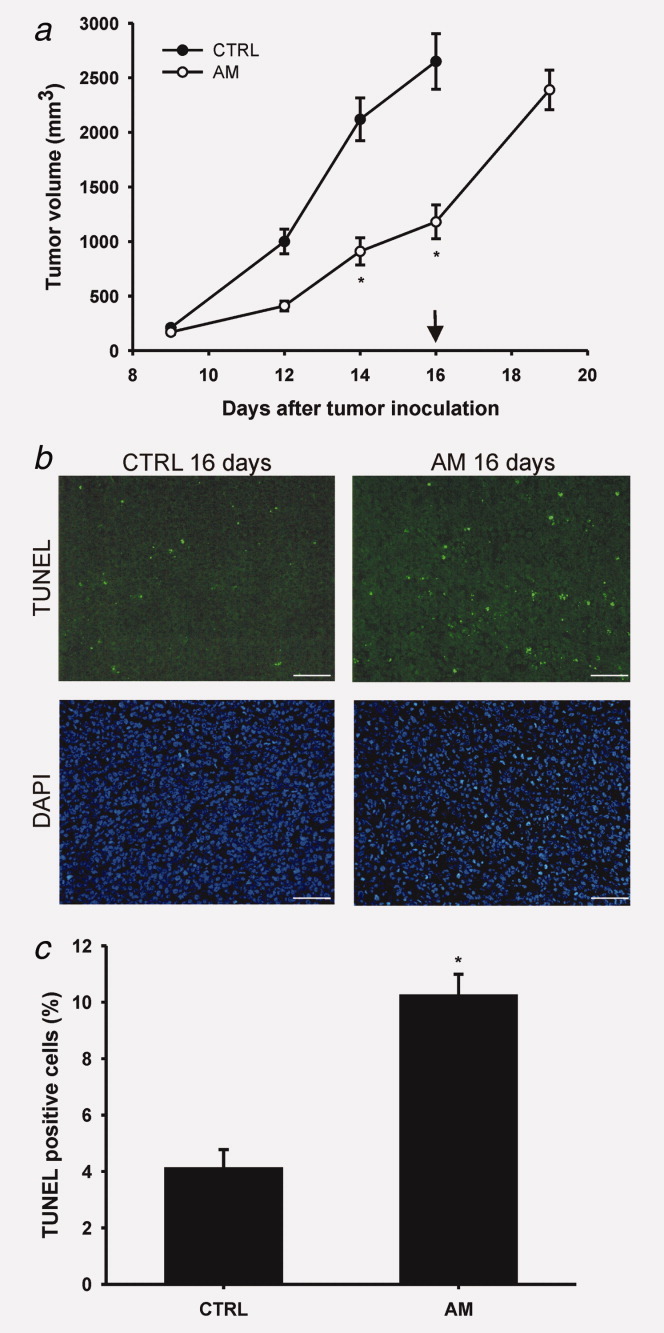
Treatment with AM inhibits tumor growth and induces apoptosis in the PC-3 human prostate carcinoma model. (*a*) Mice were treated with AM started from the first day after tumor inoculation. Arrow indicate the last day of treatment. Error bars represent SEM. *p < 0.001 (Student's *t*-test). (*b*) Representative images of TUNEL assay. Bars represent 100 μm. (*c*), Percentage of TUNEL positive cells. Data are presented as mean ± SEM of nine sections. *p < 0.001 (Student's *t*-test).

To correlate our *in vitro* findings with the *in vivo* antitumor activity of the AM we performed an *in vivo* experiment in which we assessed the extent of apoptosis in the tumor tissue with a TUNEL assay. We have found that treatment of PC-3 tumor xenografts with AM produced a 55% TGI at the end of the 16 days long treatment period (Fig. 3*a*), and the number of apoptotic cells in the treated group showed a 2.5-fold increase compared with the control group (Figs. 3*b* and 3*c*).

### The AM reduces the mitochondrial membrane potential (ΔΨ_m_), decreases mitochondrial mass, and activates caspase-9

**Figure 4:**
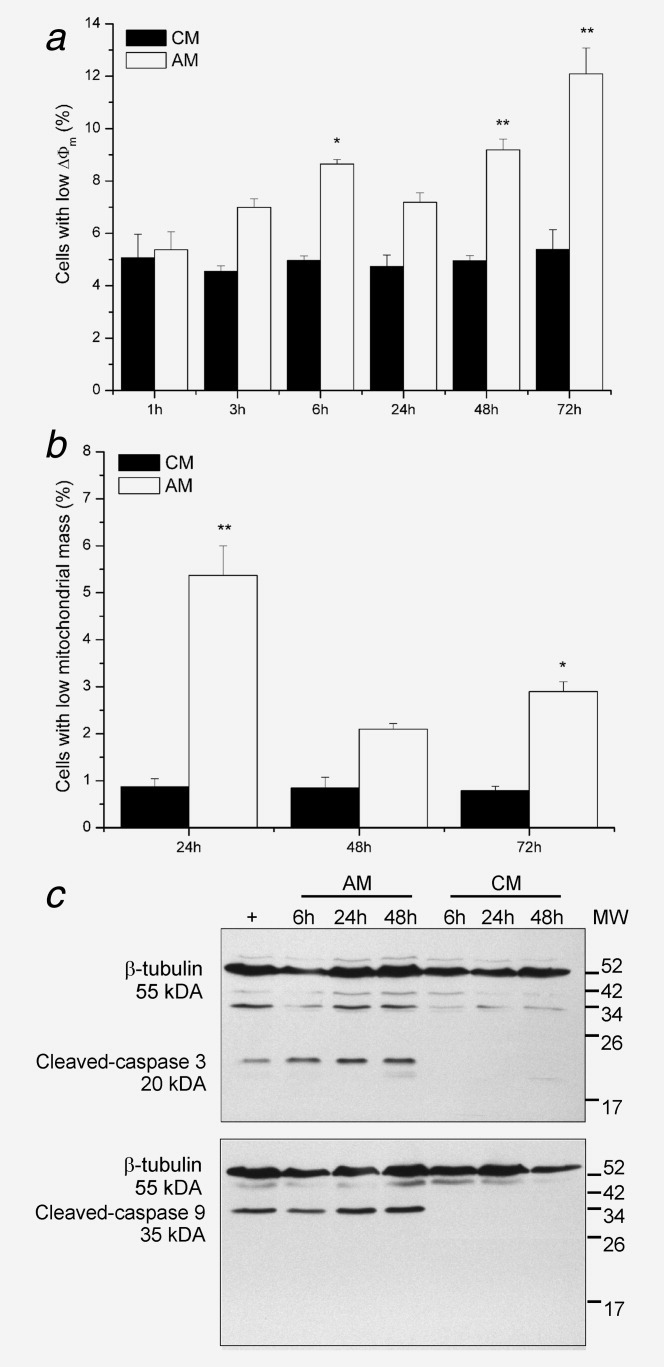
AM activates the mitochondrial pathway of apoptosis in PC-3 cells. (*a*) Percentage of cells with low ΔΨ_m_ as measured by JC-1. *p < 0.05, **p < 0.001 (ANOVA). (*b*) Percentage of cells with decreased mitochondrial mass as measured by NAO. *p < 0.001 (ANOVA). (*c*) Western-blot analysis of protein lysates from cells treated with AM or CM. + Lysate from Jurkat cells treated with 25 μM etoposide. Data are presented as mean ± SEM of three independent experiments (panels *a* and *b*) or representative of three independent experiments (panel *c*).

To study the mechanism of apoptosis induction we investigated the effect of AM on ΔΨ_m_ and mitochondrial mass in PC-3 cells. We found that treatment with AM significantly increased the percentage of cells with low ΔΨ_m_ as measured by JC-1 (Fig. 4*a*), and with low mitochondrial mass as measured by acridine orange 10-nonyl bromide (NAO) (Fig. 4*b*). To further examine the mechanism of apoptosis we investigated whether caspase-3, 8 and 9 are activated. According to our results caspase-9 and caspase-3 are activated after 6–24 hr of treatment (Fig. 4*c*), while we found no evidence of the processing of caspase-8 (data not shown).

### The AM inhibits proliferation and induces G1 arrest in PC-3 cells

**Figure 5:**
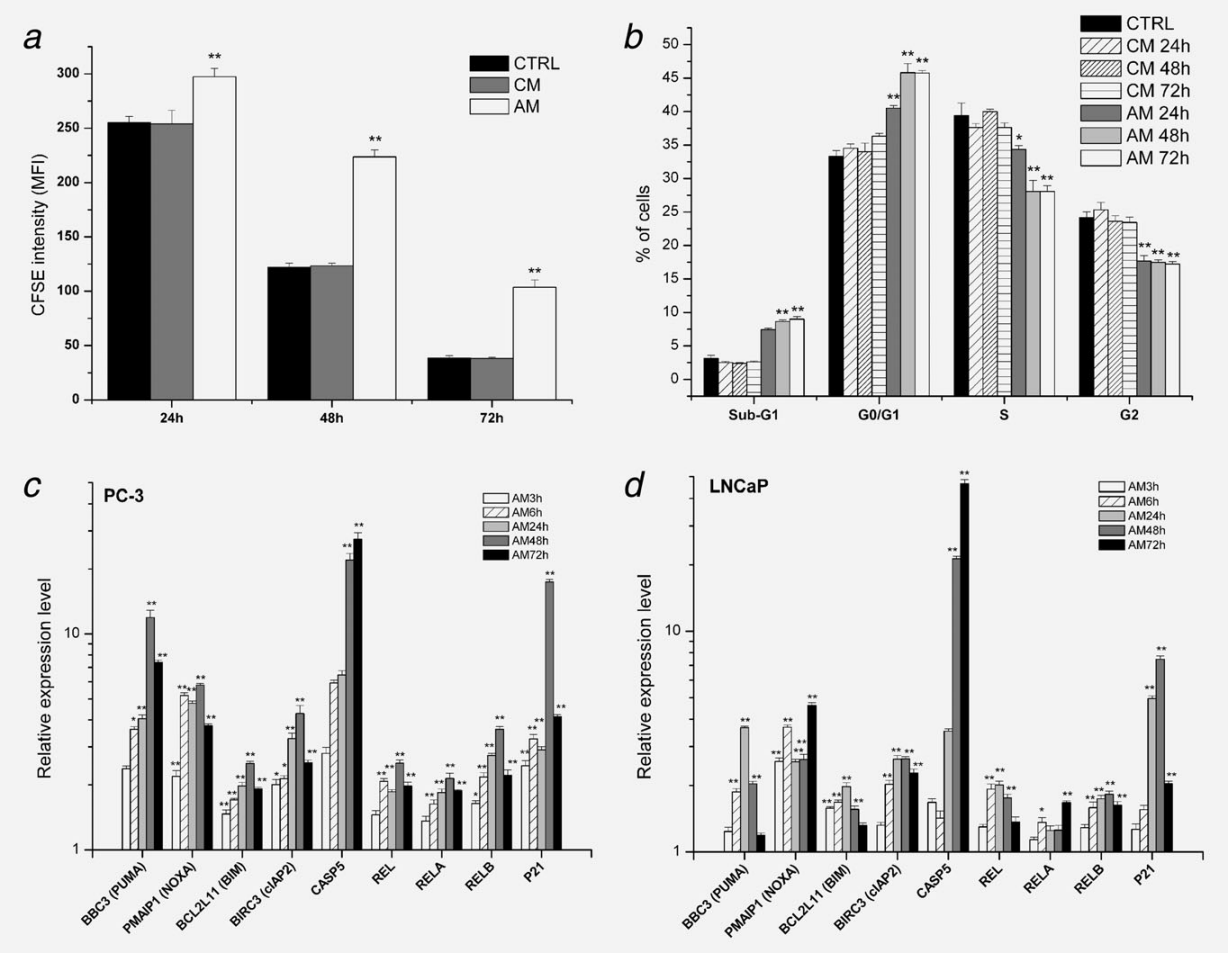
AM inhibits proliferation, induces G1 arrest, and influences gene expression in PC-3 cells. (*a*) Cell division tracking of CM or AM treated cells with CFSE. The mean fluorescence intensity (MFI) is plotted against the treatment lengths. (*b*) Cell cycle analysis of cells treated with AM using propidium iodide staining. (*c*) Quantitative RT-PCR analysis of gene expression in AM treated PC-3 cells. (*d*), As in *c*, but LNCaP cells were treated. Data are presented as mean ± SEM of three independent experiments. *p < 0.05, **p < 0.001 (ANOVA).

To further explore the mechanism of cell growth inhibition we performed cell division tracking with the dye carboxyfluorescein succinimidyl ester (CFSE). We found that treatment with AM significantly inhibited the dilution of CFSE (Fig. 5*a*). The average doubling time calculated from the kinetics of the intensity decay was increased from 20.6 ± 2.8 hr (CM treated cells) to 28.6 ± 4.0 hr (AM treated cells).

Next we examined whether the slower rate of cell division is associated with alterations in the cell-cycle phase distribution. Cell-cycle analysis showed that treatment with AM for 24 hr causes G1-arrest, which is accompanied by a decrease in the percentage of cells in S and G2/M phase (Fig. 5*b*). The percentage of cells with hypodiploid DNA content (sub-G1) was increased after 48 and 72 hr of treatment (Fig. 5*b*). These results suggest that upon treatment with AM the cells are transiently arrested in G1; however, additional experiments are needed to clarify whether the apoptotic cells originate from the G1-arrested population or become apoptotic during the S/G2 transition. The CM had no influence on the cell cycle distribution of PC-3 cells.

### The AM influences the expression of genes involved in apoptosis and cell cycle regulation

Next we measured the transcript levels of certain proapoptotic genes, anti-apoptotic genes, genes involved in NF-κB signaling, and genes regulating the cell cycle by QPCR after various periods of treatment with AM or CM. Since the expression of many of the above genes is regulated by P53, these experiments were carried out in both PC-3 (mutated p53) and LNCaP (wild type p53) cells. Treatment of PC-3 cells with AM increased the expression of the BH3-only protein encoding *PUMA* (11.9-fold, *p* < 0.001), *NOXA* (5.7-fold, *p* < 0.001), and *BIM* (2.5-fold, *p* < 0.05) compared with untreated control (Fig. 5*c*). Interestingly, the expression level of the anti-apoptotic protein encoding *cIAP2* seemed to be also increased (4.3-fold, *p* < 0.001). Among the caspase encoding genes only the expression level of *CASP5* was increased (27.4-fold, *p* < 0.001), while among the NF-κB signaling related genes the expression level was increased in the case *REL* (2.5-fold, *p* < 0.001), *RELA* (2.1-fold, *p* < 0.001), and *RELB* (3.6-fold, *p*< 0.001). The expression level of *CDKN1A* was increased to 17.4-fold (*p* < 0.001) (Fig. 5*c*). Treatment with CM had no effect on the expression of genes investigated (data not shown). Essentially similar results were obtained when LNCaP cells were treated with AM (Fig. 5*d*).

## Discussion

In this study we demonstrated that a mixture of amino acids, vitamins and other small molecules (AM) has antitumor activity in both murine and human xenograft tumor models. Our *in vivo* studies show that AM inhibits the growth of Colon 26 murine colorectal adenocarcinoma, B16 murine melanoma, MXT murine mammary carcinoma, S180 murine sarcoma, P388 murine lymphoid leukemia, HL-60 human promyeloid leukemia, PC-3 human prostate carcinoma and HT-29 human colon carcinoma with a TGI ranging from 40 to 69%.

We have shown earlier that the AM, but not the individual components induced apoptosis of tumor cell lines.^19^ Here we showed that the AM is capable to inhibit the growth of a wide range of murine and human tumors, induces apoptosis of PC-3 human prostate carcinoma cells *in vitro*, and increases the number of apoptotic cells in PC-3 xenografts. The fact that the antitumor activity of the AM could also be demonstrated in CB17/ICR-*Prkdc*^*scid*^ mice indicates that the tumor inhibitory effect of the AM is not dependent on functional T and B cells. It has been reported that the inhibition of arginase I and l-arginine supplementation inhibits the growth of Lewis lung carcinoma, however the inhibitory effect could not be demonstrated in immunodeficient mice.^25^ Thus the mechanism of the *in vivo* antitumor effect of the AM seems to be different from that of arginase I inhibition. These data support a direct tumor inhibitory effect of the AM, though participation of the components of innate immune mechanisms which are functional despite of the *scid* mutation could not be excluded. Thus further experiments performed with the same syngeneic tumor model and identical administration schedule using various immunodeficient and immunocompetent hosts are needed to clarify the role of immune mechanisms in the antitumor activity of the AM.

According to our results the antitumor activity of the AM is not dependent on P53 function, since inhibition of tumor growth could also be demonstrated in tumor models which have deleted or mutated *P53* gene (P388, HL-60, HT-29). Moreover, the AM induced apoptosis in PC-3 cells and xenografts, which are reported to have a frame shift mutation in the *P53* gene.^26^

The tumor growth inhibitory effect observed in human tumor xenografts seemed to be slightly lower when compared with the growth inhibition measured in syngeneic mouse tumors (Table 1). This could be explained by the different treatment schedules: in case of the human xenografts treatment was initiated when the tumors became detectable at Day 7 after inoculation, while treatment in the mouse tumor models was started at Day 1 after tumor inoculation. This caused a shift in the ratio of AM components to tumor mass, which in case of the human xenografts resulted in a lower relative amount of AM components. The decrease of the relative amount of AM was more pronounced when the mice had two tumors/animal and the treatment was initiated when the tumors became detectable. Consequently, the effect of AM decreased slightly further but it remained significant (Supporting Information Fig. S2). This is in agreement with our results which indicate that the antitumor activity of the AM is dose dependent. In addition, when treatment of PC-3 xenografts has been initiated on the first day after tumor inoculation the inhibition of tumor growth was comparable with the growth inhibition observed in the mouse tumor models (Fig. 3*a*).

We have previously shown that combination of AM and various cytostatic agents (doxorubicin, etoposide, mitoxantrone, 5-FU, vinblastine, mitomycin and cytarabine) increases the *in vitro* inhibitory effect on the growth of a number of tumor cell lines (K562, Jurkat, A20, MCF7, HeLa).^21^ Here we demonstrated that the AM has a comparable antitumor effect with 5-FU and cisplatin in the Colon-26 tumor model. Moreover, combination of AM and 5-FU or cisplatin enhances the *in vivo* tumor growth inhibitory effect, which could provide rationale for the combined use of AM and cytostatic agents in clinical practice. Furthermore, the tumor growth inhibitory effect of the AM proved to be sustainable over a long-term treatment period, which in our view also supports the potential use of AM in cancer therapy.

To further investigate the mechanism of tumor growth inhibition we performed additional *in vitro* experiments with AM on PC-3 cells. It seems that the AM induces apoptosis via the mitochondrial pathway, since treatment with AM causes mitochondrial depolarization, decreases the mitochondrial mass, and activates caspase-9 and caspase-3. However, according to our results inhibition of cell proliferation and/or changes in the cell-cycle distribution could also be involved in the cell growth inhibitory effect of the AM.

To gain further insight into the mechanism of apoptosis induction and G1 arrest we quantified the expression level of selected genes. According to our results treatment with AM increases the expression of *PUMA*, *NOXA* and *BIM*. These pro-apoptotic members of the Bcl-2 family are among the effectors of mitochondrial apoptosis and considered to be primary targets of P53; however, P53-independent induction of these genes is also reported.^27^, ^28^ This is in agreement with our findings, which show that the expression levels of these genes were elevated in both PC-3 and LNCaP cells. The induction of these genes supports our functional data, and these results collectively point toward the induction of apoptosis through the mitochondrial pathway. Surprisingly the expression level of the anti-apoptotic gene *cIAP2* was also elevated in both cell lines. Though this finding is seemingly contradictory to the apoptosis inducing effect of the AM, it is possible that the transcriptional activation of *cIAP2* is compensated by the elevated expression of the pro-apoptotic genes, and the net effect is the apoptosis of the cell. This hypothesis is supported by the work of Bednarski et al.^29^ who showed that doxorubicin induces *cIAP2* in sarcoma cells, while the net effect of doxorubicin treatment was the apoptosis of the sarcoma cells (though in their case the compensatory effect was due to the downregulation of other anti-apoptotic genes). The elevated expression of the cyclin-dependent kinase inhibitor *CDKN1A* supports the G1-arrest causing effect of the treatment with AM. Elevated *CDKN1A* expression was found in both cell lines tested, which is consistent with the fact that *CDKN1A* could be induced either in a P53-dependent or a P53-independent fashion.^30^ These *in vitro* findings collectively indicate that treatment with AM in addition to apoptosis induction through the mitochondrial pathway also slows the proliferation rate and causes G1 arrest in PC-3 cells. However, further experiments are needed to clarify the functional relationship of these effects.

The selective accumulation of amino acids and other small molecules by cancer cells in theory provides a possibility to interfere with the metabolic activity of the malignant cells by decreasing the availability of the substances which are taken up in increased amounts. Indeed there are reports which demonstrate that the restriction of tyrosine, methionine and phenylalanine availability affects the invasion related signaling pathways, modulates the metastatic phenotype, causes cell cycle arrest, and induces apoptosis in melanoma and prostate cancer cell lines *in vitro.*^31^–^33^ In addition the *in vivo* metastasis or tissue infiltration inhibiting effect of tyrosine and phenylalanine restriction has also been demonstrated in murine melanoma, leukemia, lung carcinoma and hepatocarcinoma models.^34^–^36^ These *in vivo* data are not necessarily in contradiction with our results, since they seem to demonstrate the inhibition of the metastatic processes, while the AM appear to exert a direct antitumor effect through the induction of apoptosis. We think that their and our studies represent two different approaches to the problem using the same starting point, namely that the accumulation of many substances is increased by cancer cells. In theory both their depletion and our “overloading” strategy could be valid.

In conclusion, we demonstrated that the AM has an *in vivo* antitumor effect, and the induction of apoptosis through the mitochondrial pathway plays a role in this tumor growth inhibitory effect. In addition to apoptosis induction the AM also slows the proliferation and induces G1 arrest of PC3-cells. The strength of the antitumor effect of the AM is comparable with that of cytostatic agents, and the combined treatment inhibits tumor growth more effectively than the single treatments. These findings together suggest that the usage of AM might offer an interesting perspective for new therapies in the treatment of cancer without side effects and in combination with other treatments may offer hope for a more effective cancer therapy.
