# Supplementary material for: A mixture of amino acids and other small molecules present in the serum suppresses the growth of murine and human tumors in vivo
Source: Int J Cancer. 2012 Aug 1;132(5):1213–21. doi: 10.1002/ijc.27756 (PMC3562491; doi:10.1002/ijc.27756)
Supplement: Supplementary file 1 [file ijc0132-1213-SD1.pdf]

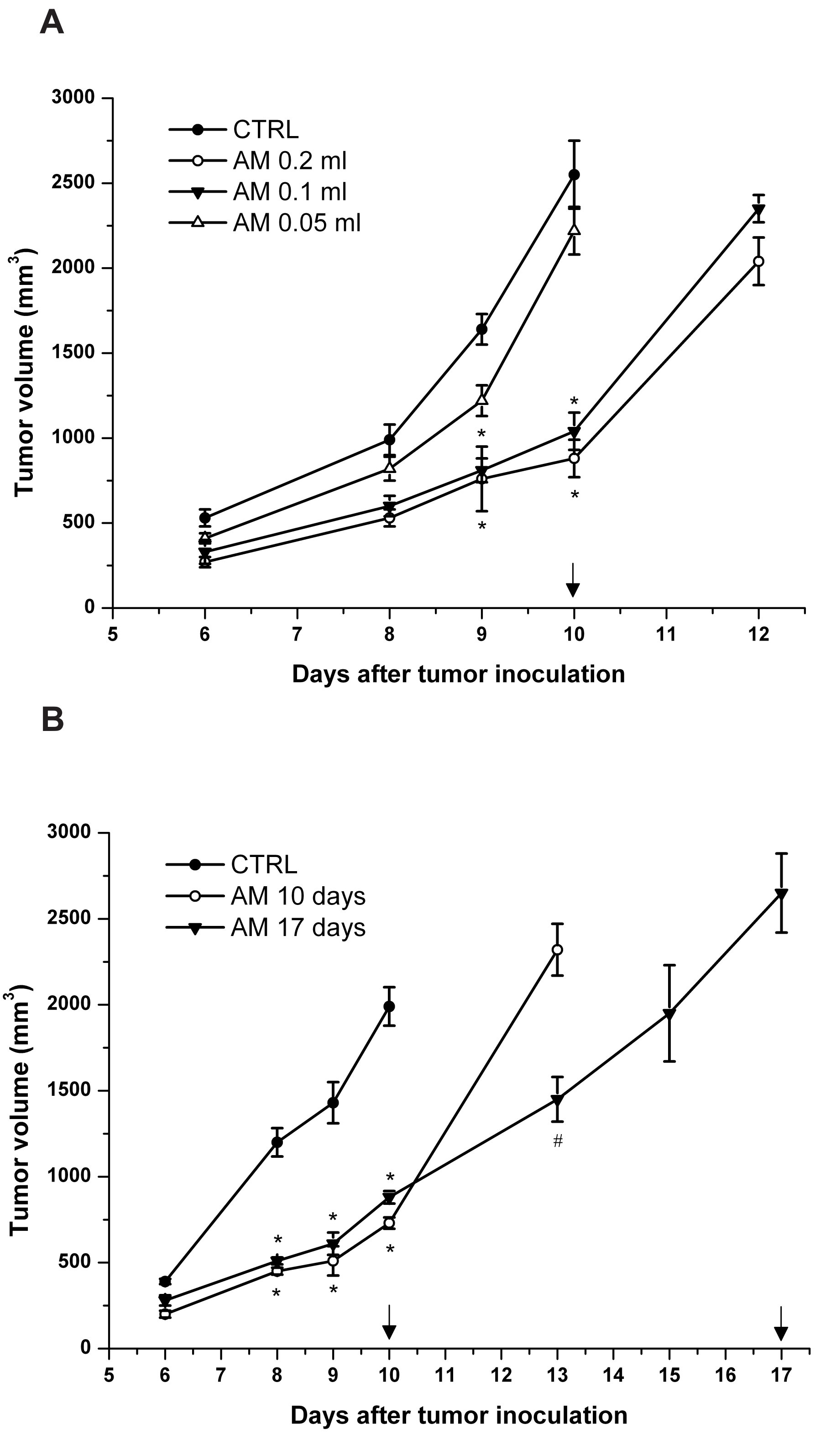

Supporting Information Figure S1. The antitumor activity of the AM is dose dependent and sustainable in the P388 lymphoid leukemia model. A, Tumor bearing mice were treated with the indicated amounts of AM. *, $P < 0.001$ vs. control (ANOVA). B, Tumor bearing mice were treated with AM for the indicated time. *, $P < 0.001$ vs. control; #, $P < 0.001$ vs. AM 10 days (ANOVA). Treatments were started on the first day after tumor inoculation. Arrows indicate the last days of treatment. Error bars represent SEM.
