# Supplementary material for: A mixture of amino acids and other small molecules present in the serum suppresses the growth of murine and human tumors in vivo
Source: Int J Cancer. 2012 Aug 1;132(5):1213–21. doi: 10.1002/ijc.27756 (PMC3562491; doi:10.1002/ijc.27756)
Supplement: Supplementary file 2 [file ijc0132-1213-SD2.pdf]

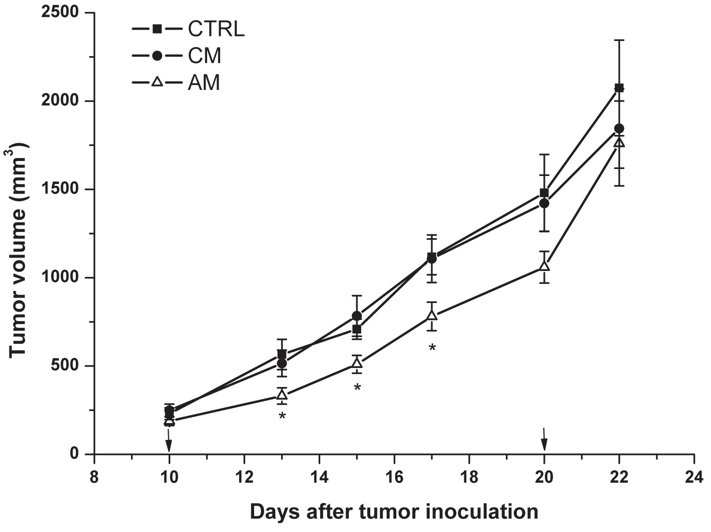

Supporting Information Figure S2. The CM has no effect on the growth of PC-3 xenografts. Mice were treated with AM or CM started from the 10th day after tumor inoculation. Error bars represent SEM. *$P < 0.05$ (Student's t-test). Arrows indicate treatment initiation and termination. Error bars represent SEM.
