# Supplementary material for: A mixture of amino acids and other small molecules present in the serum suppresses the growth of murine and human tumors in vivo
Source: Int J Cancer. 2012 Aug 1;132(5):1213–21. doi: 10.1002/ijc.27756 (PMC3562491; doi:10.1002/ijc.27756)
Supplement: Supplementary file 3 [file ijc0132-1213-SD3.pdf]

A

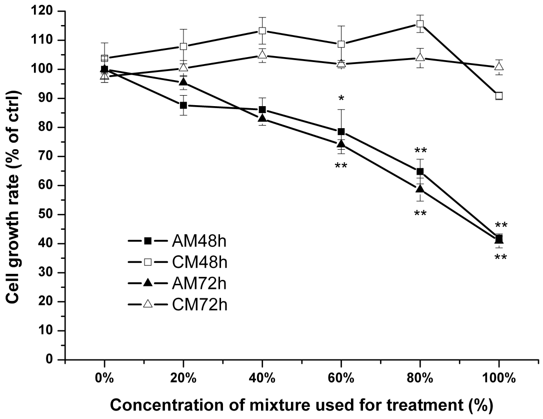

B

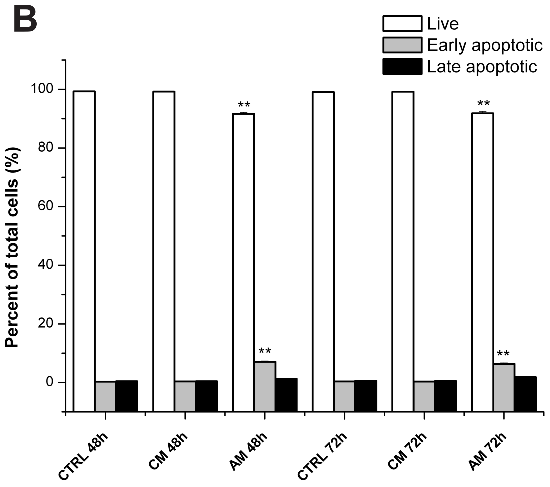

Supporting Information Figure S3.The AM inhibits the growth and induces apoptosis of human prostate carcinoma PC-3 cells in vitro. A, The effect of 48h or 72h treatment with the indicated concentration of CM or AM on cell proliferation as measured by WST-1 assay. * $P < 0.05$; ** $P < 0.001$ (ANOVA). B, Percentage of live (Annexin V^-/PI^-), early apoptotic (Annexin V^+/PI^-) and late apoptotic (Annexin V^+/PI^+)cells after treatment with CM or AM. ** $P < 0.001$ (ANOVA). Data are presented as mean ± SEM of three independent experiments.
