# Supplementary material for: A mixture of amino acids and other small molecules present in the serum suppresses the growth of murine and human tumors in vivo
Source: Int J Cancer. 2012 Aug 1;132(5):1213–21. doi: 10.1002/ijc.27756 (PMC3562491; doi:10.1002/ijc.27756)
Supplement: Supplementary file 4 [file ijc0132-1213-SD4.pdf]

Supporting Information Table 1. Primers used for real-time PCR amplification of selected genes.		
**Gene Symbol**	**Forward Primer (5'-3')**	**Reverse Primer ((5'-3')**
APAF1	TGCGCTGCTCTGCCTTCT	CATGGGTAGCAGCTCCTTCTTC
BAD	GCTGTGCCTTGACTACGTAACATC	TGCTCACTCGGCTCAAACTCT
BAK1	CACGGCAGAGAATGCCTATGA	CCCAATTGATGCCACTCTCA
BAX	GGTTGTCGCCCTTTTCTACTTTG	CAGTTCCGGCACCTTGGT
BCL2	CCGCATCAGGAAGGCTAGAG	CTGGGACACAGGCAGGTTCT
BCL2L1	GATTGCCTTTGTTTTGATGTTTGT	GGAAAGGGAACCCAGGTTAGTG
BCL2L11	GCCGCCACTACCACCACTT	AACCGAATACCGCGATGATG
BID	GCTGTATAGCTGCTTCCAGTGTAGA	GCTATCTTCCAGCCTGTCTTCTCT
BIK	TGATGGAGACCCTCCTGTATGAG	AGAGTCAGTCATGCCAAGAACCT
BIRC2	AGTAACTGGGAACCAAAGGATGAT	CATGTGTCTGCATGCTCAGATTT
BIRC3	TGGTTTCCAAGGTGTGAGTACTTG	GGGCTGTCTGATGTGGATAGC
BIRC5	CCCCTCGGGCCAACTG	GTTTGGCTTGCTGGTCTCTTCT
BIRC6	AGCCGAAGGATAGCGATCAG	TAGCCCGGAAGACGAAGAAA
CASP1	TCACTGCTTCGGACATGACTACA	GGAACGTGCTGTCAGAGGTCTT
CASP2	TGAGCTGTGACTACGACTTGAGTCT	GTCGACAGGCGGAGCTTCT
CASP3	CAGTGGAGGCCGACTTCTTG	ATGAACCAGGAGCCATCCTTT
CASP4	TTTCCTGGCAATTGAAAATGG	AAGGTGCTCCTTGAAGTTGATTAAG
CASP5	CGGAAGGTACAGAAATCATTTGAA	GTCAAGGTTGCTCGTTCTATGGT
CASP6	TGGATGCAGCCTCCGTTTAC	TCCCGGTGAGAATAATATCCTTCT
CASP7	CGGAGCGACGGAGAGAGA	AACCAACACAGTCCATTACAGCAT
CASP8	CTTTGACCACGACCTTTGAAGAG	GTGGTCCATGAGTTGGTAGATTTTC
CASP9	ACTGGCTCCAACATCGACTGT	TTCACCTCCACCATGAAATGC
CASP10	GGCCCTCATTCCCATTCG	GGTTCAGAGCATCTGCTTCGA
CASP14	TCGAAAGCACCATGAAAAGAGA	GGCCTGCTGGAATTTTTCC
CDKN1A	GCGGCAGACCAGCATGA	ATTAGGGCTTCCTCTTGGAGAAG
CDKN2A	GCCTTTTCACTGTGTTGGAGTTT	CGCAAGAAATGCCCACATG
CHUK	CGGGCCCTGGGAGATG	ATCGTTCTCTGTTTTTGGTACTTAGCT
DAPK1	GCTGCTGAGGAGCTTTTTCAG	GGTGGGAACCTGCTGGAGTT
DIABLO	AATCACATTCAGCTGGTGAAACTG	TGCCAGCTTGGTTTCTGCTT
IKBKB	TCTGCGCTTAGATACCTTCATGAA	CTTGCTGCAGGACGATGTTTT
IKBKE	CTCGCCGCCTCCCATAG	CCCCTCGCAGAGCTCTTG
IKBKG	CTGTCCCAAGTGCCAGTATCAG	CCGGCCCTACTCAATGCA
NFKB1	GGCTACACCGAAGCAATTGAA	CAGCGAGTGGGCCTGAGA
NFKBIA	GCTGAAGAAGGAGCGGCTACT	TCGTACTCCTCGTCTTTCATGGA
NFKBIB	TCATCAGCATGAACCCTTCCT	GGTCATTCTGCAGGTCCATGT
NFKBIE	CCCGTTCCTCACCTTCGA	CTGGTTTATTGCCCCATGCT
NFKBIZ	ACCAGGCTTCCCTGTACCAGTA	CCAGAGTTGGTTTGTGGGTGTA
REL	GCCCATCTCAAGTGGATTGTC	GCCACTGATGACCAGCTTGAA
RELA	GCCGGGATGGCTTCTATGA	TGGATTCCCAGGTTCTGGAA
RELB	GACCCCCATGGCATCGA	CGTGGTTGGGCAGGAAGT
XIAP	GGAGTGCAATGGCACAATCTT	CCGTGGAGCATGCCTGTAGT
