# Supplementary material for: A mixture of amino acids and other small molecules present in the serum suppresses the growth of murine and human tumors in vivo
Source: Int J Cancer. 2012 Aug 1;132(5):1213–21. doi: 10.1002/ijc.27756 (PMC3562491; doi:10.1002/ijc.27756)
Supplement: Supplementary file 5 [file ijc0132-1213-SD5.doc]

**Supporting Information Materials and Methods**

**Materials**

The selection process of components of the AM and CM has been described previously (1, 2). On the basis of literary data and theoretical considerations, the most important of which were the selective accumulation of certain small molecules by cancer cells and the hypothesis of an anti-tumor defense mechanism formed by the small substances of the circulatory system which are accumulated by the tumor cells (3), we selected L-tryptophan, L-tyrosine, L-methionine, L(-)malate and L-ascorbate. First we determined by preliminary experiments those non-toxic concentrations of these substances one by one in which they had no effect on the growth of the Sp2/0‑Ag14 mouse myeloma cells *in vitro* when applied alone. Then we used these concentrations in the combination experiments, and found that these five substances when applied in combination could inhibit the growth of the cancer cells. In the next step we tested another 84 substances occurring in the circulatory system (all amino acids, nucleobasis, nucleosides, monosaccharides, vitamins and membrane permeable intermediates) and found that 11 out of the 84 (L-phenylalanine, L-arginine, L-histidine, deoxy-D-ribose, d-biotin, pyridoxine, adenine, riboflavin, D(+)-mannose, hippuric acid, and orotic acid) could potentiate the growth inhibitory effect of the initial five component mixture described above We considered the 73 molecules which could not potentiate the growth inhibitory effect of the initial five component mixture as ineffective.

On the basis of these results a “practical” AM has been formulated which contains 12 of the identified 16 effective components, and this “practical” AM has been used in the experiments presented in this paper (see main text).

The CM contains 12 of the 73 ineffective compounds, with similar physiological characteristics (6 amino acids, 4 vitamins, 1 monosaccharide, and 1 nucleobase.) as components of the AM, at concentrations ensuring the same osmolarity as the AM.

**Cell lines and tumors**

The Colon 26 adenocarcinoma cells, B16 melanoma solid tumors, MXT hormone sensitive mammary carcinoma solid tumors, S180 sarcoma solid tumors, P388 lymphoid leukemia cells, HL-60 human promyeloid leukemia cells, HT-29 human colon carcinoma cells, were obtained from the Division of Cancer Treatment and Diagnosis (DCTD), National Cancer Institute, NIH (Frederick, MD). The PC-3 human prostate carcinoma cells and the LNCaP human prostate carcinoma cells were obtained from the ECACCC. Cells were maintained in RPMI 1640 medium supplemented with 10% FCS. The cells were subcultured weekly and were routinely monitored for mycoplasma contamination with MycoSensor PCR Assay Kit (Stratagene, Budapest, Hungary). Tumors were established or maintained by serial passage in the appropriate strains of mice, and were cryopreserved for further experiments.

# Animals

Eight weeks old BD2F1 female mice (C57BL female x DBA/2 male), BALB/c female mice, and CB17/ICR-*Prkdc^scid^* male mice were obtained from the colonies housed in specified pathogen free (SPF) environment at the Department of Experimental Pharmacology, National Institute of Oncology (Budapest, Hungary). The animals were kept on ventilated racks in macrolon cages at 22-24 ^o^C (50-60% humidity), with a 12 h day/night light cycle. The animals had free access to tap water and were fed with a sterilized standard diet (Charles River, Isaszeg, Hungary) *ad libitum*. The breeding stock was controlled (pathology-parasitology, hystology, bacteriology, and serology) by the Central Veterinary Institute (Budapest, Hungary). Seven mice were treated in each group and each experiment was repeated at least two times, with the exception of the single experiment with CM on PC-3 xenografts where two tumors were implanted into each mice and five mice were treated in each group.

**Cell growth assay**

Cells were plated at a density of 5x10^3^ cells/well in 96-well plates, and were treated with different concentrations of AM or control mixture for 48h or 72 h in quintuplicate. The number of viable cells was measured with WST-1 cell proliferation reagent (Roche, Budapest, Hungary). After washing with PBS, 100 µl of phenol red free RPMI medium containing 10 µl of WST-1 reagent was added to each well. Cells were incubated at 37 ºC for 30 min, and the optical density was measured at 450 nm with an EL800 microplate reader.

**Annexin V staining and flow cytometry**

Cells were plated into 6 well plates at a density of 10^5^ cells/well. After treatment with AM or control mixture for 72 h cells were detached with trypsin, washed in cold PBS, and stained with the Annexin V-FITC Apoptosis Detection Kit (eBioscience, Vienna, Austria) in 100 µl binding buffer containg 5 µl Annexin V-FITC. Following incubation at room temperature for 15 min in the dark, samples were diluted to 1 ml with binding buffer, and propidium iodide (PI) was added to a final concentration of 0.5 µg/ml. Samples were run on a FACSCalibur cytometer and 2x10^4^ events were analyzed using the CellQuest software (Becton Dickinson, Pécs, Hungary)

**TUNEL assay**

10 µm thick frozen sections were cut from 3 tumors of each experimental group. From each tumor specimen three sections, separated by 1 mm distance, were prepared and analyzed by DeadEnd Fluorometric TUNEL System (Promega, Budapest, Hungary) according to the manufacturer’s instructions. In brief, the sections were fixed in 4% paraformaldehyde for 15 min, and digested with 20 µg/ml proteinase K for 10 min. After washing with PBS the sections were incubated with reaction buffer containing terminal deoxynucleotidyl transferase and fluorescein-12-dUTP at 37 ºC for 60 min. The reaction was stopped with 2xSSC (300 mM NaCl, 30 mM sodium-citrate, pH 7.0), then the sections were washed three times with PBS, and the nuclei were counterstained with 0.5 µg/ml 4',6-Diamidino-2-phenylindole (DAPI) in PBS for 2 min. The acquisition of digital images was performed using the analySIS software with a CCD camera connected to an Olympus BX61 fluorescent microscope at 20x magnification. Image analysis was performed with ImageJ software (4). Three fields form each section were included in the image analysis and the number of fluorescein positive nuclei was normalized to the number of DAPI positive nuclei.

**Measurement of mitochondrial membrane potential and mitochondrial mass**

Mitochondrial membrane potential (ΔΨ_m_) was measured with MitoProbe JC-1 Assay kit (Invitrogen, Budapest, Hungary) according to the manufacturer’s instructions. In brief, cells were detached with non-enzymatic cell dissociation solution, resuspended in 1 ml RPMI medium, and incubated with 2 µM 5,5,6,6-tetrachloro-1,1,3,3-tetraethyl-benzamidazolylcarbocyanine iodide (JC-1) at 37 ºC for 15 min. To the positive control samples carbonyl cyanide 3-chlorophenylhydrazone (CCCP) was added to a final concentration of 100 µM. After washing, the samples were run on a Becton-Dickinson FACSCalibur cytometer and 2x10^4^ events were analyzed using the CellQuest software. Cells with decreased ΔΨ_m_ were defined as events with high FL1 (green) and low FL2 (red) fluorescence intensity.

For the measurement of the mitochondrial mass cells were detached as above and were incubated with 10 μM acridine orange 10-nonyl bromide (NAO, Invitrogen, Budapest, Hungary) for 15 min at 37ºC. Samples were measured as described above, and the percentage of cells with decreased FL1 fluorescence intensity was calculated.

**Western blot**

Cells were lysed in ice cold buffer containing 10 mM TRIS HCL pH 7.4, 10 mM NaCl, 3 mM MgCl2, 1 mM EDTA, 0.1% NP40, 1 mM DTT and Proteoblock protease inhibitor cocktail (Fermentas, Szeged, Hungary). Protein concentration was measured with DC protein assay (Biorad, Budapest, Hungary). 150 µg protein/well was separated on a 15% SDS-polyacrylamide gel and transferred to nitrocellulose membranes. After blocking with 5% non-fat dry milk (Cell Signaling, Budapest, Hungary) in wash buffer (136mM NaCl, 20mM Tris-base pH 7.6, 0.1% Tween 20) for 1 h, membranes were incubated overnight with primary antibodies (Cleaved-caspase 3: 1:250 dilution, Cleaved-caspase 9: 1:500 dilution, β-tubulin: 1:1000 dilution in 5% BSA containing wash buffer; Caspase 8 antibody was diluted 1:200 in blocking buffer). After washing, peroxidase-conjugated anti-rabbit antibody diluted at 1:2000 in blocking buffer was added for 1 hour. Membranes were developed with Immobilon Western ECL HRP substrate kit (Millipore, Szeged, Hungary) and exposed to x-ray films. The polyconal antibodies recognizing cleaved-caspase 3, cleaved-caspase 9, β-tubulin and the peroxidase-conjugated anti-rabbit antibody were obtained from Cell Signaling (Budapest, Hungary). The antibody recognizing both inactive and active forms of caspase 8 was obtained from Santa Cruz (Budapest, Hungary).

**Cell division tracking**

Cells were labeled with carboxyfluorescein succinimidyl ester (CFSE) at a density of 10^6^ cell/ml in serum free RPMI medium for 15 min. The labeling was quenched by addition of 5 volumes of RPMI with 10% FCS. Cells were washed twice with RPMI containing 10% FCS and were plated at a density of 5x10^5^ cells/flask. Treatments were started on the next day and the CFSE intensity (FL1) was quantified at 24h intervals with flow cytometry using a FACSCalibur cytometer. 6x10^4^ events were analyzed using the CellQuest software. The cell doubling time was calculated from the decay kinetics of CFSE intensity.

**Cell cycle analysis**

Cells were trypsinized, fixed in 70% ethanol at 4 °C overnight, and washed once with PBS. The cells were incubated for 60 min at room temperature in 0.5 ml of phosphate-citric acid buffer (0.2 M NaHPO4, 0.1 M citric acid, pH 7.8). After centrifugation the cells were resuspended in 1 ml of propidium iodide solution (1% Triton X-100, 80 μg/ml propidium iodide, and 0.1 μg/ml DNase-free RNase A). After a 60 min incubation in the dark the DNA content was analyzed by acquisition of 3x10^4^ events using a FACSCalibur cytometer and CellQuest software. The cell cycle distribution was quantified using the Weasel software.

**Quantitative RT-PCR (QPCR)**

Total RNA was isolated with RNeasy Plus Mini Kit (Qiagen, Gödöllő, Hungary) and was treated with DNase I. cDNA was prepared with Verso SYBR Green 2-step QRT-PCR Rox kit (Abgene, Budapest, Hungary). PCR primers used for real-time quantitative amplification of the housekeeping genes human 18S ribosomal RNA, Hprt1 and cyclophilin A were described previously (5). PCR primers for human APAF1, BAD, BAK1, BAX, BCL2, BCL2L1, BCL2L11, BID, BIK, BIRC2, BIRC3, BIRC5, BIRC6, CASP1-CASP10, CASP14, CDKN1A, CDKN2A, CHUK, DAPK1, DIABLO, IKBKB, IKBKE, IKBKG, NFKB1, NKFB2, NFKBIA, NFKBIB, NFKBIE, NFKBIZ, , REL, RELA, RELB, XIAP were designed by Primer Express Software (Applied Biosystems, Budapest, Hungary) (Supporting Information Table 1). The expression levels of BBC3 and PMAIP1 were measured with TaqMan gene expression assays (BBC3: Hs00248075_m1, PMAIP1: Hs00560402_m1, HPRT: Hs02800695_m1 from Applied Biosystems, Budapest, Hungary). PCR reactions were run in triplicates using Absolute QPCR SYBR Green ROX Mix (Abgene, Budapest, Hungary) or using TaqMan gene expression master mix (Applied Biosystems, Budapest, Hungary ) on an ABI StepOne Real Time PCR System (Applied Biosystems, Budapest, Hungary). The stability of the expression level of the housekeeping genes was analyzed and Hprt1 was chosen for normalization of target gene expression. The relative gene expression levels were calculated by dividing the normalized target gene expression measured in the treated samples by that of the untreated control samples. Statistical analysis was performed separately on the expression data of individual genes comparing the relative expression levels at the different time points with ANOVA followed by Bonferroni test.

**References**

1. Kulcsár G. Inhibition of the growth of a murine and various human tumor cell lines in culture and in mice by mixture of certain substances of the circulatory system. Cancer Biother 1995;10:157-76.
2. Kulcsár G. Synergistic potentiating effect of D(+)-mannose, orotic, and hippuric acid sodium salt on selective toxicity of a mixture of 13 substances of the circulatory system in culture for various tumor cell lines. Cancer Detect Prev 2000;24:485-95.
3. Kulcsár Gy. Theoretical and literary evidence for the existence of the passive antitumor defense system. Cancer Biother Radiopharm 1997;12: 281-6.
4. Rasband WS. ImageJ. U.S. National Institutes of Health, Bethesda, Maryland, USA, http://rsb.info.nih.gov/ij/, 1997-2009.
5. Colell A, Ricci JE, Tait S, Milasta S, Maurer U, Bouchier-Hayes L, Fitzgerald P, Guio-Carrion A, Waterhouse NJ, Li CW, Mari B, Barbry P, Newmeyer DD, Beere HM,Green DR. GAPDH and autophagy preserve survival after apoptotic cytochrome c release in the absence of caspase activation. Cell. 2007;129:983-97.
